# Molecular diagnosis of hereditary inclusion body myopathy by linkage analysis and identification of a novel splice site mutation in *GNE*

**DOI:** 10.1186/1471-2350-12-87

**Published:** 2011-06-28

**Authors:** Steven E Boyden, Anna R Duncan, Elicia A Estrella, Hart GW Lidov, Lane J Mahoney, Jonathan S Katz, Louis M Kunkel, Peter B Kang

**Affiliations:** 1 Program in Genomics, Division of Genetics, Children's Hospital Boston, 300 Longwood Avenue, Boston, MA 02115, USA; 2 Department of Genetics, Harvard Medical School, 77 Avenue Louis Pasteur, Boston, MA 02115, USA; 3 Department of Pathology, Children's Hospital Boston, 300 Longwood Avenue, Boston, MA 02115, USA; 4 Department of Pathology, Harvard Medical School, 77 Avenue Louis Pasteur, Boston, MA 02115, USA; 5 California Pacific Medical Center Research Institute, P.O. Box 7999, San Francisco, CA 94120, USA; 6 The Manton Center for Orphan Disease Research, Children's Hospital Boston, 300 Longwood Avenue, Boston, MA 02115, USA; 7 Department of Neurology, Children's Hospital Boston, 300 Longwood Avenue Boston, MA 02115, USA; 8 Department of Neurology, Harvard Medical School, 300 Longwood Avenue Boston, MA 02115, USA

## Abstract

**Background:**

Many myopathies share clinical features in common, and diagnosis often requires genetic testing. We ascertained a family in which five siblings presented with distal muscle weakness of unknown etiology.

**Methods:**

We performed high-density genomewide linkage analysis and mutation screening of candidate genes to identify the genetic defect in the family. Preserved clinical biopsy material was reviewed to confirm the diagnosis, and reverse transcriptase PCR was used to determine the molecular effect of a splice site mutation.

**Results:**

The linkage scan excluded the majority of known myopathy genes, but one linkage peak included the gene *GNE*, in which mutations cause autosomal recessive hereditary inclusion body myopathy type 2 (HIBM2). Muscle biopsy tissue from a patient showed myopathic features, including small basophilic fibers with vacuoles. Sequence analysis of *GNE *revealed affected individuals were compound heterozygous for a novel mutation in the 5' splice donor site of intron 10 (c.1816+5G>A) and a previously reported missense mutation (c.2086G>A, p.V696M), confirming the diagnosis as HIBM2. The splice site mutation correlated with exclusion of exon 10 from the transcript, which is predicted to produce an in-frame deletion (p.G545_D605del) of 61 amino acids in the kinase domain of the GNE protein. The father of the proband was heterozygous for the splice site mutation and exhibited mild distal weakness late in life.

**Conclusions:**

Our study expands on the extensive allelic heterogeneity of HIBM2 and demonstrates the value of linkage analysis in resolving ambiguous clinical findings to achieve a molecular diagnosis.

## Background

Hereditary inclusion body myopathy (HIBM) is characterized by slowly progressive muscle weakness, preferentially affecting the tibialis anterior and usually sparing the quadriceps. Onset is generally between the ages of 20 and 40 and serum creatine kinase (CK) levels are normal or minimally elevated. Histological features include the presence in myofibers of vacuoles rimmed with basophilic granular material, as well as cytoplasmic filamentous inclusions on electron microscopy [1]. Rimmed vacuoles are a defining characteristic of HIBM, but are also observed less consistently in other muscle disorders [2], including limb girdle muscular dystrophy (LGMD) types 1A [3] and 2G [4]. The autosomal recessive HIBM type 2 (HIBM2) is caused by mutations in the gene encoding glucosamine (UDP-*N*-acetyl)-2-epimerase/*N*-acetylmannosamine kinase (*GNE*) [5], and is allelic with distal myopathy with rimmed vacuoles (DMRV), also known as Nonaka myopathy (NM) [6-10].

We present a family with multiple siblings affected with a distal myopathy with vacuolated myofibers. Linkage analysis excluded known genes for recessive forms of LGMD and many other muscle disorders, but directed our attention to *GNE *as a likely candidate. The identification of compound heterozygous mutations in *GNE*, including a novel splice site mutation, confirmed the diagnosis as HIBM2.

## Methods

Written informed consent for participation in this study was obtained for all subjects, in accordance with the Institutional Review Board of Children's Hospital Boston. Patient confidentiality was protected in accordance with the Health Insurance Portability and Accountability Act of 1996. Saliva samples were collected using the Oragene·DNA kit (DNA Genotek) and genomic DNA was isolated. Seven family members were genotyped at 10,204 single nucleotide polymorphisms using the GeneChip Human Mapping 10 K 2.0 Xba Array (Affymetrix). Genomewide multipoint parametric linkage scans were performed using MERLIN v1.1.2 [11]. The disease allele frequency was set to 0.0001 and we used a full penetrance, zero phenocopy model. Marker map positions and Caucasian allele frequencies were provided by Affymetrix. The error checking and Pedwipe functions of MERLIN were used to remove unlikely genotypes. Amplification of candidate gene exons and splice junctions by polymerase chain reaction (PCR) and sequencing of purified products were performed by standard protocols, and sequence data were analyzed using Sequencher v4.8 (Gene Codes) and SeqScape v2.5 (Applied Biosystems). The c.1816+5G>A mutation was genotyped in DNA samples from unrelated control subjects using a Custom TaqMan SNP Genotyping Assay (Applied Biosystems). Multi-species sequence alignments were performed in ClustalW [12]. Mutation positions were numbered relative to RefSeq transcript NM_005476.5 and isoform NP_005467.1.

Muscle biopsy tissue was available for the proband, and total RNA was isolated using the RNeasy Fibrous Tissue Mini Kit (Qiagen). Additional saliva samples were collected from the proband's parents using the Oragene·RNA kit (DNA Genotek) and total RNA was isolated using the RNeasy Mini kit (Qiagen). The c.1816+5G>A splice site mutation was characterized by reverse transcriptase PCR (RT-PCR) using the SuperScript III One-Step RT-PCR System (Invitrogen) and primers designed to amplify exons 7 through 12. RT-PCR products were screened by the QIAxcel automated capillary electrophoresis system (Qiagen). Selected products were subjected to agarose gel electrophoresis and individual bands were excised, gel-purified using the QIAquick Gel Extraction Kit (Qiagen), and sequenced.

## Results

### Case reports

**Figure 1:**
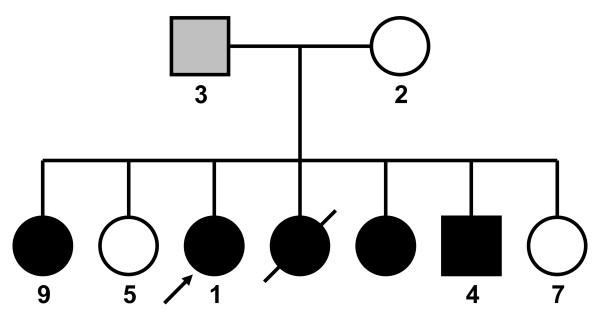
**Pedigree**. Subjects with DNA samples available and tested are labeled with their identification number. Black-filled symbols represent individuals affected with HIBM2, with onset in their 20s or 30s. Tested patients were compound heterozygous with genotype NM_005476.5: c.[1816+5G>A]+[2086G>A]. The gray-filled symbol represents an individual presenting in his 70s with mild HIBM-like symptoms, with genotype NM_005476.5: c.[1816+5G>A]+[=]. Arrow indicates the proband.

**Figure 2:**
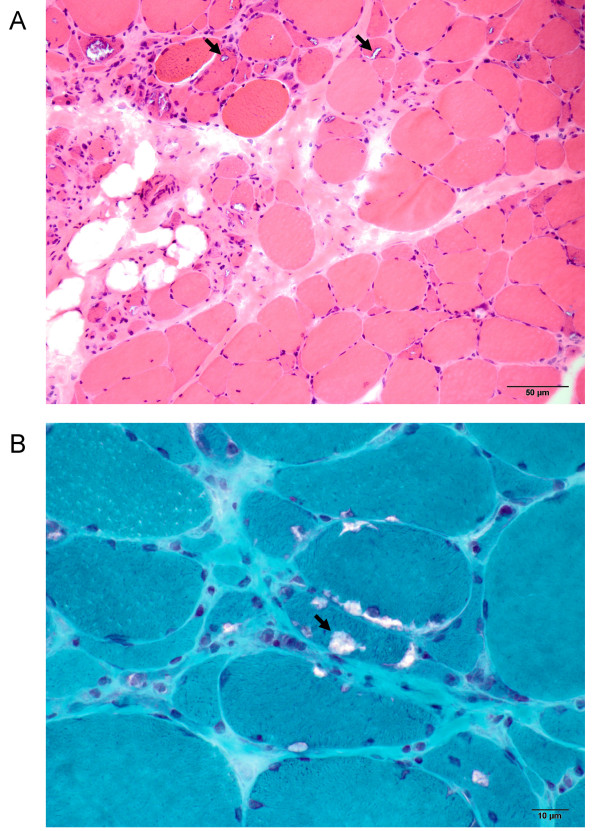
**Pathology characteristic of HIBM in biceps muscle biopsy from proband**. A) Hematoxylin and eosin staining showed marked variation in fiber diameter, rounded fibers, mild endomysial fibrosis, slight fatty infiltration, and irregular vacuoles (arrows) containing granular material. Frankly necrotic fibers, basophilic regenerating fibers, and inflammatory infiltrates were not prominent. The disease process appeared focal, with fibers in some areas mildly affected, while other regions, including the one shown here, exhibited severe myopathic pathology. B) Modified Gomori trichrome staining highlighted small amounts of granular red material within and around the vacuoles (arrow), as well as endomysial fibrosis and small, angulated, atrophic fibers.

The family consisted of five affected siblings, two unaffected siblings, their unaffected mother, and their father, who had mild distal weakness noted late in life (Figure 1). The female proband presented at age 37 with weakness, fatigue while walking, and difficulty walking and climbing stairs. Her weakness was predominantly in the distal lower extremities, which showed mild atrophy. She exhibited quadriceps sparing, as knee extension strength was 5/5 bilaterally on the Medical Research Council scale, while knee flexion and hip flexion strength were 2/5 bilaterally. Ankle dorsiflexion and eversion were 1/5 on the right side and 0/5 on the left. The distal upper extremities were less severely affected. Electromyography data were unavailable. Progression was slow and after more than 15 years, she remained ambulant with assistance at age 53. Serum CK was mildly elevated at 370 U/L, and there was no evidence of cardiac, respiratory, or non-muscular neurological involvement. A first biopsy taken from the left quadriceps was reported as normal. Subsequent biopsy of biceps tissue revealed pathology that was strikingly focal; affected areas showed vacuoles with thin basophilic rimming, as well as marked variation in fiber size, rounded fibers, infiltration of endomysial tissue, increased internalized nuclei, and abundant atrophic fibers (Figure 2). On clinical histological examination, cytoplasmic bodies were seen, though rarely, and the vacuoles did not stain positive for amyloid by Congo red.

Three of the proband's sisters were reported as affected, with onset in their 30s, but additional clinical information was unavailable. An affected brother presented at age 27 with inability to stand on tiptoe, followed later by difficulty running and walking. His progression was relatively rapid; by age 33 he began to fall and lost ambulation, whereas his four affected sisters remained ambulant with assistance at ages ranging from 39 to over 50. At the time of examination, the brother had lost nearly all movement in his legs; his weakness was too advanced to assess quadriceps sparing. He was rated as 1/5 for knee extension, knee flexion, and hip flexion strength, and 0/5 for ankle dorsiflexion and eversion. He could not stand from sitting and had severe problems with activities of daily living. A few years later he developed weakness of his upper extremities that was initially proximal but later distal, manifesting as weak grip and difficulty writing. Electromyography was not performed. Serum CK was mildly elevated at 420 U/L (reference range 57-366), and there was no cardiac, respiratory, or non-muscular neurological involvement. For both the proband and her affected brother, the diagnosis was reported at different exams as either possible limb girdle muscular dystrophy or a distal myopathy.

The proband reported both of her parents to be asymptomatic, making the mode of inheritance appear autosomal recessive, but later her father was noted to exhibit slowness in walking and a shuffling gait. Upon clinical evaluation, the mother of the proband was unaffected, and the proband's father (age 79 at exam) had full strength in his upper extremities and proximal lower limbs. However, his ankle dorsiflexion was described as weak and rated as 4+/5, and he was unable to heel-walk, indicating possible weakness of the tibialis anterior muscles. He also had difficulty toe-walking. His symptoms mimicked the pattern of weakness of his five affected children, but were markedly less severe. The age of onset of his symptoms was unknown.

### Linkage scan and DNA sequence analysis

The parents of the proband originated from the same region of India, but were not known to be related. The proportion of homozygous and heterozygous genotypes in their children, compared to children of known consanguineous and non-consanguineous couples, supported the hypothesis that the parents were unrelated (data not shown). A linkage scan under an autosomal recessive model produced fourteen linkage peaks at or near the maximum possible LOD score of 1.454. The only known LGMD gene within any of these linkage peaks was *CAV3*, in which mutations cause autosomal dominant LGMD1C [13]. *CAV3 *was screened and no mutations were found. A linkage peak on chromosome 9 contained three genes associated with different muscle disorders: *VCP*, *TPM2*, and *GNE*. Mutations in *VCP *and *TPM2 *were considered unlikely due to the mode of inheritance, clinical presentation, or pathological characteristics of the associated diseases not matching those of the family [14,15]. However, review of the patients' clinical and histological information suggested their presentation was consistent with a diagnosis of HIBM.

Sequence analysis of *GNE *revealed the proband was heterozygous for both a novel mutation in the 5' consensus splice donor sequence of intron 10 (c.1816+5G>A) and a known missense mutation in exon 12 (c.2086G>A, p.V696M), previously reported in families of Indian and Thai origin [5,16-18]. The mutations segregated according to the disease in all available family members and were confirmed to be in trans; the father was heterozygous for c.1816+5G>A, the mother was heterozygous for c.2086G>A (p.V696M), all three available affected children were compound heterozygous for both mutations, and the two unaffected children were both heterozygous for one of the two mutations. The novel c.1816+5G>A mutation was absent from 703 unrelated control subjects comprised of 446 of Middle Eastern ancestry and 257 of miscellaneous European ancestry. The c.1816+5G nucleotide was conserved in 18 of 20 vertebrate species examined; two species of bird had a cytosine at that position (Additional file 1: Supplemental Figure S1).

Because the proband's carrier father showed very mild myopathic symptoms and her brother was more severely affected than his affected siblings, we sequenced the gene *N*-acetylglucosamine kinase (*NAGK*) in these two individuals to search for a potential modifier allele. NAGK has been reported to be partially functionally redundant with GNE kinase activity in some model systems [19-23]. However, there were no mutations in the coding sequence or splice junctions in either subject.

### Mutation characterization

**Figure 3:**
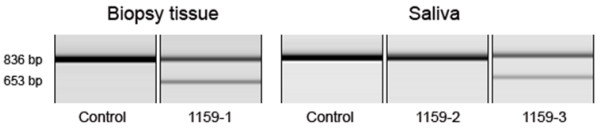
**Correlation of *GNE *transcript length with c.1816+5G>A splice mutation genotype**. RNA was extracted from muscle biopsy tissue for subject 1159-1 and from saliva for subjects 1159-2 and 1159-3. By capillary electrophoresis, RT-PCR produced a single band of the expected size in control samples and 1159-2, the mother of the proband, in whom the c.1816+5G>A mutation was absent. The proband (1159-1), and her father (1159-3), both of whom were heterozygous for the c.1816+5G>A mutation, showed the full length band and a shorter band that lacked exon 10.

An RT-PCR amplicon spanning exons 7 to 12 of the *GNE *transcript showed two products in the proband and her father, both of whom carried the c.1816+5G>A splice site mutation, whereas the proband's mother and control samples showed only the expected full-length band (Figure 3). Both bands were individually purified and sequenced from the proband and her father, and in both subjects the larger product was confirmed to represent the full-length amplicon, while the smaller product lacked exon 10. Skipping of exon 10, comprised of 183 base pairs, at the mRNA level would result in an in-frame deletion (p.G545_D605del) of 61 amino acids. Both the p.G545_D605del and p.V696M mutations are in the kinase domain of the GNE protein; mutations throughout the gene, in both the epimerase and kinase domains, can be pathogenic for HIBM2 [5].

## Discussion

We report a family with five siblings affected with HIBM2, which we diagnosed primarily by linkage analysis and identification of mutations in *GNE*. Recently, we used a similar approach to achieve a molecular diagnosis for a family with multiminicore disease, another rare myopathy that can be mistaken for LGMD [24]. The exclusion in our linkage scan of almost all known myopathy genes [25], despite fourteen regions showing linkage at a LOD score of approximately 1.4, confirms the value of linkage analysis as a diagnostic tool even in families not informative enough to generate suggestive or significant LOD scores.

Several features of the clinical and pathological presentation of the family made the diagnosis initially uncertain. First, the proband came to medical attention prior to the identification of mutations in *GNE *as the cause of HIBM, so genetic testing for HIBM was not then available. Moreover, the symptoms were suggestive of LGMD, and LGMD is much more prevalent than HIBM [26-30]. HIBM is predominantly found among Middle Eastern Jewish families [5,31] and Japanese families (as DMRV/NM) [10,32]; it appears to be much rarer among other populations [33]. Only a few previous families of Asian Indian ancestry have been documented with mutations in *GNE *[5,16]. Furthermore, an initial biopsy of the quadriceps of the proband appeared normal, which in retrospect is consistent with a quadriceps-sparing myopathy, but may have initially confounded diagnosis. A later biopsy revealed the pathologic hallmarks of HIBM; however, the characteristic rimmed vacuoles are not specific to HIBM [2] and were negative for amyloid by Congo red staining, which is atypical for HIBM [34]. Finally, because five of seven siblings were affected and their father appeared symptomatic, autosomal dominant inheritance was plausible, provided that the father's much milder symptoms could be explained by variable expressivity. Therefore, both dominant and recessive muscular dystrophies and myopathies were potential candidates.

Our linkage scans were performed under a recessive model, and our identification of compound heterozygous mutations in affected subjects confirmed this mode of inheritance. However, the father (Subject 3) was heterozygous for the c.1816+5G>A splice site mutation, and at age 79 he displayed slight weakness in his lower extremities similar in its pattern to his affected children. The predicted deletion of 61 amino acids (p.G545_D605del) in the kinase domain of GNE would be expected to result in an extremely hypomorphic or null allele, and thus the weakness in Subject 3 could possibly be a result of haploinsufficiency of *GNE*. Mutations showing incomplete dominance have been documented for other recessive myopathies [35-37]. However, without additional clinical examination and biopsy tissue we could not determine if the symptoms in Subject 3 represented a milder version of HIBM or normal age-related weakness. Alternatively, an unknown enhancer mutation in a modifier gene, or an effect of gender on the manifestation of the splice mutation, could potentially account for the myopathic symptoms in Subject 3 as well as the faster deterioration of his affected son than his four affected daughters.

The mechanism whereby mutations in *GNE *result in muscle weakness is not completely understood [38]. Approximately 80 different mutations in *GNE *have been previously reported [18,33,39-42], of which only two are in splice sites. Like the c.1816+5G>A mutation described herein, these mutations likely result in null alleles [10,43]. The vast majority of mutations in *GNE *are missense [33], and no patients have been recorded as homozygous or compound heterozygous for two null mutations, suggesting a complete loss of function of *GNE *might be lethal in humans [5], as in mice [44]. Accordingly, the p.V696M mutation reported both here and previously [5,16-18], often in conjunction with very severe mutations, may be relatively benign, as otherwise the patients might not retain enough residual *GNE *activity to permit life.

## Conclusions

We demonstrate the utility of genetic analyses as a diagnostic tool for heterogeneous disorders. In a family with an undiagnosed distal myopathy, we excluded known candidate genes by linkage analysis and identified compound heterozygous mutations in *GNE*, which, in conjunction with biopsy analysis, confirmed the diagnosis as hereditary inclusion body myopathy type 2. A novel splice site mutation, c.1816+5G>A, resulted in skipping of exon 10 and a predicted in-frame deletion in the kinase domain of GNE, likely producing a null allele. A c.1816+5G>A carrier showed mild and late-onset distal weakness, so further study to evaluate the effect of partial *GNE *function may be warranted. Our data contribute to a more complete description of the clinical and allelic heterogeneity of HIBM2, and may facilitate diagnosis in other unresolved cases.

## Competing interests

The authors declare that they have no competing interests.

## Authors' contributions

SEB performed linkage analysis, sequenced some candidate genes, genotyped controls, and wrote the manuscript. ARD sequenced most of the candidate genes and performed RT-PCR. EAE recruited the family, collected saliva samples, and obtained consents. HGWL photographed and interpreted slides of muscle biopsy tissue. LJM sequenced a candidate gene. JSK performed clinical examinations of some of the affected siblings and their parents. LMK supervised the project and provided financial support. PBK conceived and managed the project, provided financial support, and edited the manuscript. All authors read and approved the final manuscript.

## Pre-publication history

The pre-publication history for this paper can be accessed here:

http://www.biomedcentral.com/1471-2350/12/87/prepub

## Supplementary Material

Additional file 1 **Supplemental Figure S1. Evolutionary conservation of the *GNE*:c.1816+5G nucleotide**. DNA sequence orthologous to human *GNE *exon 10 and intron 10 was aligned in 20 vertebrate species, of which 18 species shared the c.1816 + 5G nucleotide. Two species of bird had a cytosine at that position. Box indicates mutated nucleotide. Bold indicates exonic sequence. Asterisks denote perfectly conserved positions. Click here for file
