# Supplementary figures and images for: Molecular diagnosis of hereditary inclusion body myopathy by linkage analysis and identification of a novel splice site mutation in GNE
Source: BMC Med Genet. 2011 Jun 28;12:87. doi: 10.1186/1471-2350-12-87 (PMC3141630; doi:10.1186/1471-2350-12-87)

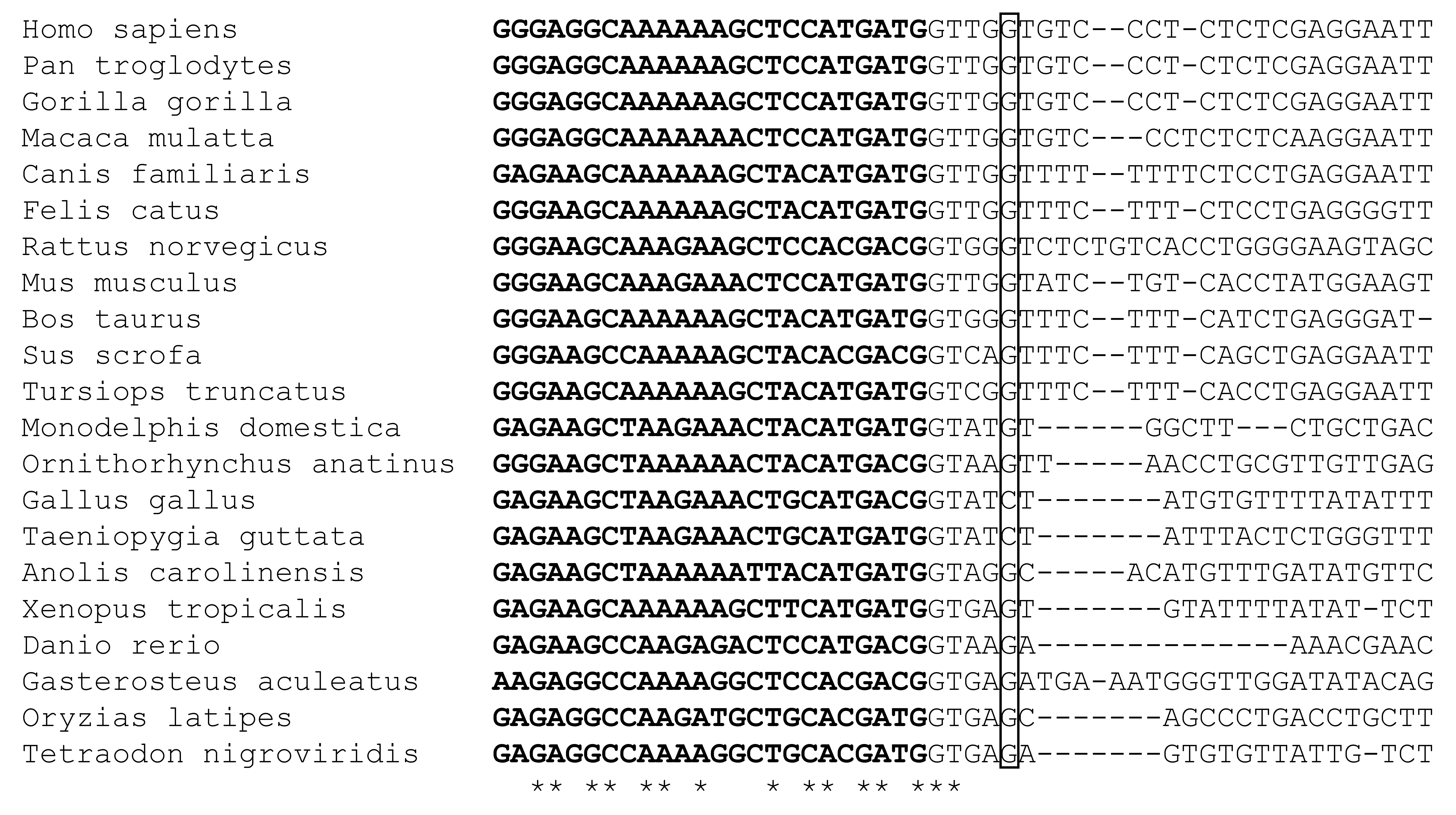

Supplement: Additional file 1 — Supplemental Figure S1. Evolutionary conservation of the GNE:c.1816+5G nucleotide. DNA sequence orthologous to human GNE exon 10 and intron 10 was aligned in 20 vertebrate species, of which 18 species shared the c.1816 + 5G nucleotide. Two species of bird had a cytosine at that position. Box indicates mutated nucleotide. Bold indicates exonic sequence. Asterisks denote perfectly conserved positions. [file 1471-2350-12-87-S1.TIFF]
